# Probiotic mediated intestinal microbiota and improved performance, egg quality and ovarian immune function of laying hens at different laying stage

**DOI:** 10.3389/fmicb.2023.1041072

**Published:** 2023-01-24

**Authors:** Hengyong Xu, Yuxiang Lu, Dan Li, Chaoyang Yan, Yuru Jiang, Zhi Hu, Zhipeng Zhang, Ranran Du, Xiaoling Zhao, Yao Zhang, Yaofu Tian, Qing Zhu, Yiping Liu, Yan Wang

**Affiliations:** ^1^Key Laboratory of Livestock and Poultry Multi-omics, Ministry of Agriculture and Rural Affairs, College of Animal and Technology (Institute of Animal Genetics and Breeding), Sichuan Agricultural University, Chengdu, Sichuan, China; ^2^Farm Animal Genetic Resources Exploration and Innovation, Key Laboratory of Sichuan Province, Sichuan Agricultural University, Chengdu, Sichuan, China

**Keywords:** probiotics, laying hen, egg quality, antioxidant capacity, ovarian, gut microbiota

## Abstract

In order to investigate the effects of dietary probiotics supplementation on laying performance, egg quality, serum hormone levels, immunity, antioxidant, and gut microbiota of layers at different laying stages, a total of 168 Tianfu green shell laying hens (28-day-old) were randomly divided into 2 treatments: a non-supplemented control diet (NC), and diet supplemented with 10 g/kg of probiotics, respectively. Each treatment had 6 replicates with 14 hens per replicate. The feeding trial lasted for 54 weeks. The results showed that the supplementation of probiotics significantly increased the average egg weight, improved egg quality (*p* < 0.05) and ovarian development. Meanwhile, probiotics increased the serum hormone levels of E_2_ and FSH, and antioxidant indices T-AOC and T-SOD (*p* < 0.05) of laying hens at different laying stages (*p* < 0.05), decreased the expression of proinflammatory factors including IL-1, IL-6 and TNF-*α* (*p* < 0.05). Furthermore, using 16S rRNA sequencing, we observed that the addition of probiotics increased the distribution of *Firmicutes*, *Bacteroidota* and *Synergistota* at early laying period. Meanwhile, *Bacteroidota*, *Actinobacteriota*, *Verrucomicrobiota* and *Deferribacterota* showed an increasing trend at the peak of egg production. The relative abundance of *Firmicutes*, *Desulfobacterota* and *Actinobacteriota* were significantly increased at the late laying period. Moreover, PICRUSt2 and BugBase analysis revealed that at the late laying period, the probiotics supplementation not only enriched many significant gene clusters of the metabolism of terpenoids and polyketide, genetic information processing, enzyme families, translation, transcription, replication and repair, and nucleotide metabolism, but also decreased the proportion of potential pathogenic bacteria. To sum up, these data show that the addition of probiotics not only improves the performance, egg quality, ovarian development and immune function of laying hens at different laying period, but also improves the gut microbiota of layers, thus enhances production efficiency.

## Introduction

Laying hens are known to be a kind of highly productive and powerful animals, and their reproductive performance determines the economic benefits of laying hens breeding. However, after the peak laying rate of high-intensity metabolism, laying hens gradually entered the late laying stage and occupied a long time in the whole production cycle. In the late stage of laying, laying hens were more susceptible to external factors due to the decline of ovarian function, and weakened resistance to stress and disease, which were often accompanied by low laying rate, low albumen height, poor eggshell quality and a variety of diseases (Liu et al., 2013, 2019), thus increasing the difficulty and cost of breeding. Therefore, it is necessary to adopt strategies to improve the performance and quality of layers in the late laying period, prolong the laying cycle and improve the reproductive efficiency of laying hens (Zhang et al., 2019; Guo et al., 2020). To reduce the use of drugs in farming and avoid antibiotic residues in eggs, producers rely on safe alternatives to improve the health and persistency of egg production in aged layers (Qu et al., 2018). So, dietary supplementation with prebiotics, probiotics and synbiotics to improve the performance and health of laying hens has attracted increasing attention (Liu et al., 2019; Lv et al., 2019).

Probiotics, also known as active bacterial preparation and growth enhancers, are generally defined as “live microorganisms which, when administered in adequate amounts, confer health benefits on the host” (Gerritsen et al., 2011). It has a wide range of species, which are typically classified as *Lactobacillus*, *Bifidobacterium* and Gram-positive cocci, and yeast (*Saccharomyces*) genera (Fijian, 2014), with health benefits, growth promotion, non-toxic side effects, and other characteristics. Probiotics supplementation in laying hen diets have been proven to improve hen’s performance parameters including egg production, feed conversion ratio and egg quality, disease resistance and animal welfare (Smith, 2014; Villageliu and Lyte, 2017; Khan et al., 2020; Sjofjan et al., 2021; Bindari and Gerber, 2022; Jiang et al., 2022). For instance, Macit et al. (2021) reported that supplementation of humate and probiotics into diets could improve the content of monounsaturated fatty acids in yolk, feed conversion ratio and yolk color. Yan et al. (2019) demonstrated that confirmed that dietary probiotics reduced shell-less egg production and improved bone mineralization in laying hens. Park et al. (2016) confirmed that probiotics (*Enterobacter faecium*) supplementation significantly increased laying rate, eggshell thickness and nutrient digestibility, and reduced ammonia emission of laying hens. The mechanism of probiotics improving hen performance and egg quality may be related to changes in intestinal microbial composition (Fuller, 1989; Mead, 2000). Probiotics have been shown in studies to promote the growth of non-pathogenic co-anaerobic bacteria and gram-positive bacteria while inhibiting pathogen proliferation and promoting nutrient digestion and utilization (Yeo and Kim, 1997; Mountzouris et al., 2010). Furthermore, probiotics may stimulate immune regulation in a non-inflammatory manner to preserve intestinal integrity (Murugesan et al., 2014; Waititu et al., 2014; Lee et al., 2015) and metabolic homeostasis (O’Mahony et al., 2015), and ultimately regulating host behavior (Bienenstock et al., 2015; Neijat et al., 2019). However, to date, numerous studies on probiotics in laying hen diets have focused on peak or late laying period (Mikulski et al., 2012; Abdelqader et al., 2013), and studies that directly link probiotics to changes in the gut microbiota of laying hens during early, peak and late laying period are scarce.

The gut microbiota of chicken is a diverse community of hundreds of different microbes, which was frequently influenced by factors such as nutrition, gender, age, breed, feeding style and breeding density (Pourabedin and Zhao, 2015; Ding et al., 2017; Wang et al., 2020). Previous studies have shown that intricate gut microbiota frequently affects the health and performance of chicken (Bai et al., 2021). For example, the *Clostridium* species in the chicken cecum, especially clusters IV and XIVa, are the dominant microbiota in the chicken cecum and has a significantly contributing to growth (Eeckhaut et al., 2011). Moreover, numerous studies have proposed multiple potential mechanisms by which prebiotic-mediated changes in gut microbiota may confer health benefits, such as producing antimicrobial factors (Muñoz et al., 2012), improving intestinal morphology (Pourabedin et al., 2014) and stimulating the adaptive immune system of the host (Yitbarek et al., 2012). In the comprehensive review by Gaggia et al. (2010), dietary prebiotic supplementation increased the abundance of *Lactobacillus* and *bifidobacterium* in the gut microbiota of chickens. However, the effects of probiotics supplementation on the gut microbiota of laying hens at different laying stage are poorly known. Hence, the aim of this study was to evaluate the effects of supplemental dietary probiotics on laying performance, egg quality, antioxidant capacity, serum hormone levels, expression level of reproductive and immune related genes and gut microbiota in laying hens at early laying, peak laying and late laying period.

## Materials and methods

### Animals, diets and experimental design

A total of 168 healthy 28-days-old Tianfu green shell laying hens with similar initial body weight (215.40 ± 0.15 g) were obtained from the poultry breeding farm of Sichuan Agricultural University (Ya’an, China) and then randomly divided into experimental group and control group. Each group had 6 replicates and each replicate included 14 chickens. From 28 days of age, the control group (NC) was only fed the base feed and water, while the experimental group (PC) was fed the basal diet plus the probiotics. The main components of the compound probiotics used in this study were *Bacillus subtilis* (≥1 × 10^9^ CFU/g), *Lactic Acid Bacteria* (≥2 × 10^7^ CFU/g) and *Saccharomyces* (≥1 × 10^7^ CFU/g), and were provided by Shandong Sukehan Biotechnology Co., Ltd. (Shandong, China). The dosage of probiotics should follow the commercial recommendations of the company, which is to add 10 g/kg of dietary weight to the diet. Meanwhile, the basal diets were based on the recommended nutrient content by the National Research Council (1994). All laying hens were housed in a fully enclosed chicken house and kept in wired three-level battery cages, with the room temperature kept at 22–26°C, the relative humidity at 65% and provided free access to feed and water throughout the experimental period. The photoperiod was set at 10 h (5 lux) from 17 weeks. and then increased 1 h per week until 16 h at 22 weeks. with an intensity of 10–15 lux. Meanwhile, the chickens were fed probiotics from 5 to 58 weeks of age, and the entire experimental period lasted 54 weeks. Furthermore, feces were cleaned regularly throughout the rearing period and layers were immunized according to normal immunization procedures.

### Sample collection

12 birds per treatment were randomly chosen for slaughter at the end of the three different experimental period: early laying period (23 weeks), peak laying period (27 weeks) and late laying period (58 weeks), respectively. Before slaughter, 4 mL blood samples were collected from the wing vein and were subsequently centrifuged for 10 min at 3000 r/min at 4°C to separate the serum. Serum was stored in a new 1.5 mL tube at −20°C until analyzed. Each hen was eviscerated immediately after slaughter, and the cecum segment was identified and lapped prior to removal. Cecal contents were collected and stored at −80°C until further analysis. The ovarian tissues were collected and weighted, and parts of ovarian tissue was frozen with liquid nitrogen and stored at −80°C for subsequent RNA extraction and qRT-PCR analysis.

### Laying performance and egg quality

Eggs were collected twice a day (11:00 am and 16:00 pm). The data on egg production, egg weight and unqualified eggs were recorded, and the average weekly egg production rate, average daily egg weight and broken egg rate of the whole laying cycle were calculated.

At the age of weeks 23, 27 and 58, 12 eggs from each treatment (balanced with egg weight, a total of 36 eggs per group during the experiment) were collected and the egg quality indices including egg weight (EW), egg shape index (ESI), eggshell strength (ESS), eggshell weight (ESW), eggshell thickness (ET), yolk weight (YW), yolk color (YC), albumen height (AH), and Haugh Unit (HU) were determined. The EW of individual egg were measured by an electronic scale. ESI was calculated using the formula: ESI (%) = (egg width in mm/egg length in mm) × 100. The width and length of eggs were measured by 0.01 mm Vernier calipers (Hoffmann Quality Tools Trading (Shanghai) Co., Ltd., China). ESS was measured by eggshell strength gauge (Robotmation, Tokyo, Japan). Subsequently, broken the egg and transferred the contents to a glass plate, using a trip-pod micrometer to measure the AH. The average AH at different locations (one near the yolk and the other at the end of the dense protein) was combined with egg weight to obtain the Haugh unit score for each egg according to the Haugh (1937) formula. The formula as follows: HU = 100 × log (height of albumen in mm-1.7 × EW^0.37^ in g + 7.6). Next, the egg yolks and albumen were separated and weighted on an electronic scale. To determine ESW, cleaned off any adhering albumen with water, dried them in a fume hood and weighted them. ET was measured in 3 different parts (blunt end, sharp end and middle) by a Vernier caliper. YC was evaluated according to Roche Yolk color fan (1, light yellow; 15, orange). The eggshell ratio (ESR, %) and the yolk ratio (YR, %) were calculated by ESW/EW × 100 and YW/EW × 100, respectively.

### Cholesterol analysis

After determination of egg quality, yolk samples from each replicate were separated from the broken eggs. One gram of each yolk sample was mechanically homogenized with 9 times the volume of absolution ice-cold ethanol (1:9 weight/volume) under ice bath condition and subsequently centrifuged at 2500 r/min at 4°C for 10 min. The supernatant was aspirated, and the cholesterol concentration was determined using a cholesterol assay kit according to manufacturer’s instruction (Baolai Biotechnology Co. Ltd., Yancheng, China).

### Measurement of serum hormone, apolipoprotein and antioxidant indexes

On the last day of week 23, 27 and 58, serum samples (n = 12) of per treatment groups were used to evaluate the concentration of follicle-stimulating hormone (FSH), luteinizing hormone (LH), estradiol (E_2_), vitellogenin (VTG) and very low-density lipoprotein (VLPL) by using commercially enzyme-linked immunosorbent assay (ELISA) kits obtained from Baolai Biotechnology Co., Ltd. (Yancheng, China) according to the manufacturer’s instructions. Also, the levels of total antioxidant capacity (T-AOC), total superoxide dismutase (T-SOD), methane dicarboxylic aldehyde (MDA) and glutathione peroxidase (GSH-Px) in serum were assessed using commercial ELISA kits and following the guidelines provided by the manufacturer (Baolai Biotechnology Co. Ltd., Yancheng, China).

### Follicle counts

The ovaries (*n* = 12) including large and small yellow follicles, obtained at weeks 23, 27 and 58 in the control group and the treatment group were weighted. Then, the follicles were dissected and placed on a filter paper moistened with physiological saline. The long and short axes of follicles between basement membranes were measured with Vernier calipers, and the pre-hierarchical follicles were separated into 4 groups according to their morphology and diameter, namely small yellow follicle (SYFs, 6–8 mm), large yellow follicle (LYFs, 8–10 mm), small white follicle (SWFs, 2–4 mm), and large white follicle (LWFs, 4–6 mm) (Johson, 2015). However, the diameter of other follicles >10 mm were hierarchical follicles (F1–F5). After classification, all types of ovarian follicles were counted and weighted.

### mRNA relative expression levels of genes related to reproduction and immunity

The relative expression levels including follicle-stimulating hormone receptor (FSHR), luteinizing hormone receptor (LHR), estrogen receptors alpha (ERSα), steroidogenic acute regulatory protein (StAR), bone morphogenetic protein 15 (BMP-15), anti-mullerian hormone (AMH), interleukin 1 (IL-1), interleukin 6 (IL-6), toll-like receptor 4 (TLR4), tumor necrosis factor-α (TNF-α), and cyclooxygenase-2 (COX2) were conducted by the quantitative real-time polymerase chain reaction (PCR) analysis.

TRIzol reagent (TakaRa, Dalian, China) was used to extract total RNA from ovarian tissues (*n* = 12) at 3 different periods (23, 27, and 58 week) in control group and treatment group according to manufacturer’s instructions. The resulting total RNA was reversely transcribed into cDNA using the PrimeScrip RT reagent kit (TakaRa, Dalian, China). Then, the cDNA was amplified in a CFX96-Touch™ Real-time PCR System (Bio-Rad Laboratories, CA, USA) using the SsoFastTM EvaGreen® supermix (Takara, Dalian, China). The reaction mixture (10 μl) for qPCR contained 5 μl 2 × SsoFastTM EvaGreen® supermix, 1 μl cDNA sample, 0.5 μl each of forward and reverse primers and 3 μl RNase free water. The reaction procedure as follows: 1 min at 98°C, and 38 cycles of 98°C for 15 s, annealing temperature for 20 s. The primer sequences are listed in Table 1. Each sample was run in triplicate, and the glyceraldehyde 3-phosphate dehydrogenase (GAPDH) was used as the housekeeping gene to calculate relative mRNA levels by using the 2^−△△Ct^ method.

**Table 1:** Primers used for quantitative real-time polymerase chain reaction.

Genes	Primer sequence (5′ → 3′)	Tm (°C)	Product length (bp)
GAPDH	F: ATGGGCACGCCATCACTATC	57	189
	R: TCACAAACATGGGGGCATCA		
FSHR	F: GTCTCACCTGCTTGCTGATTCTCC	50.7	109
	R: AGCTGGACCACCTTGATCTCCTG		
LHR	F: CGTCCTCATAACCAGCCACTACAAG	56.3	119
	R: TCTGAGCATCCACCGAAGCAATG		
ERα	F: TTCCGCTCTACGACCTCTTACTGG	58.3	99
	R: GGTTTCGGTTCTCCTCTTCCATTGG		
StAR	F: AGGGTTGGGAAGGACACTCTGATC	56.3	97
	R: GGGAGCACCGAACACTCACAAAG		
BMP-15	F: CTTCCTCAATGACACCCGCA	56.3	187
	R: GGGAGCGATGATCCAATGGT		
AMH	F: TCGCTCTGCTGCTCTTCTACCC	52	149
	R: CACCGAGGCTCTGAGGAGTAGG		
IL-1	F: GCCTGCAGAAGAAGCCTCG	60	203
	R: GACGGGCTCAAAAACCTCCT		
IL-6	F: AATGCCTGACGAAGCTCTCC	60	95
	R: CTCGACGTTCTGCTTTTCGC		
TLR4	F: AGTTTCCTGTCGGACTCAGC	60.4	174
	R: GTAGGCAGGTGTGTGGCATA		
TNF-α	F: CCCATCTGCACCACCTTCAT	60.4	221
	R: AACTCATCTGAACTGGGCGG		
COX2	F: CGCAATCCCTGGACGACTAA	60.4	101
	R: TGTAGCTGTGGTTAGCTCCG		

### Cecal microbial sequencing analysis

Total genomic DNA was extracted from cecal content samples at 3 different periods (21, 27, and 58 weeks) in control group and treatment group using the QIAamp Fast DNA stool Mini Kit (Qiagen, Germany) according to the procedure provided by manufacturer. The concentration and integrity of DNA was assessed by a NanoDrop 2000 spectrophotometer (Thermo Fisher Scientific, Waltham, MA, USA) and 1% agarose gel, respectively. The V3-V4 region of the 16S rRNA gene was amplified using the primer 341F (5’-CCTACGGGNGGCWGCAG-3′) and 805R (5′- GACTACHVGGGTATCTAATCC-3′) in the ABI GeneAmp 9700 PCR System. A 25 μL mixture of 12.5 μL Phusion® Hot Start Flex 2× Master Mixart version, 5.0 μL of forward and reverse primers (1 μM), and 50 ng of template DNA was prepared for PCR. Thermal cycling as follows: 98°C for 30 s, 98°C for 10 s for 35 cycles, 54°C for 30 s, 72°C for 45 s, and 72°C for 10 min. The final amplified products were purified by an AxyPrep DNA gel extraction kit (Axygen Bioscience, Union City, USA) and quantified by a Qubit dsDNA BR Assay Kit (Invitrogen, Shanghai, China). At last, the purified PCR products were sequenced on the Illumina MiSeq platform according to the standard protocols of the LC-Biotechnology Co., Ltd. (Hangzhou, Zhejiang Province, China).

### Sequence processing and bioinformatical analysis

After the original pairing sequence were demultiplexed, FLASH software (version 1.2.7) was used to merge the resulting sequences, and fastp (version 0.19.6) was used to filter the quality (Magoc and Salzberg, 2011; Chen et al., 2018). High-quality reads were de-noised using the divisive amplicon denoising algorithm (DADA2) (Callahan et al., 2016) plugin in QIIME2 (version 2020.2). RDP classification algorithm was used to classify each 16S rRNA gene sequences from the SILVA database (v138).^1^ Alpha diversity indices including Chao 1, ACE, Observed-species, Good-coverage, Shannon and Simpson were calculated using the QIIME (version 1.7.0). The beta (Bray–Curtis dissimilarity metric) diversity was analyzed by the “phyloseq” in R package. Analysis of similarities (ANOSIM) was carried out in the R package vegan. Linear discriminant analysis effect size (LEfSe) analysis was conducted to analyze the statistical significance and biological relevance. PICRUSt2 was used to predict the functional differences of cecal microbiota in chickens at different laying stages (Parks et al., 2014), and KEGG metabolic pathway was used to observe the functional differences among different groups.

### Statistical analysis

The results of laying performance, serum parameters, egg quality, and relative gene expression were all tested by one-way ANOVA using the SPSS 22.0 software (SPSS Inc., Chicago, IL, United States) followed by Duncan’s multiple comparison test. If *p* < 0.05, the difference was significant. All data in this study were presented as mean ± standard error of mean (SEM), and the graphs were generated by GraphPad Prism software 8.0 (GraphPad Inc., San Diego, USA).

## Results

### Probiotics supplementary improved hen productivity, ovarian weight and numbers of the different-sized follicles

**Figure 1:**
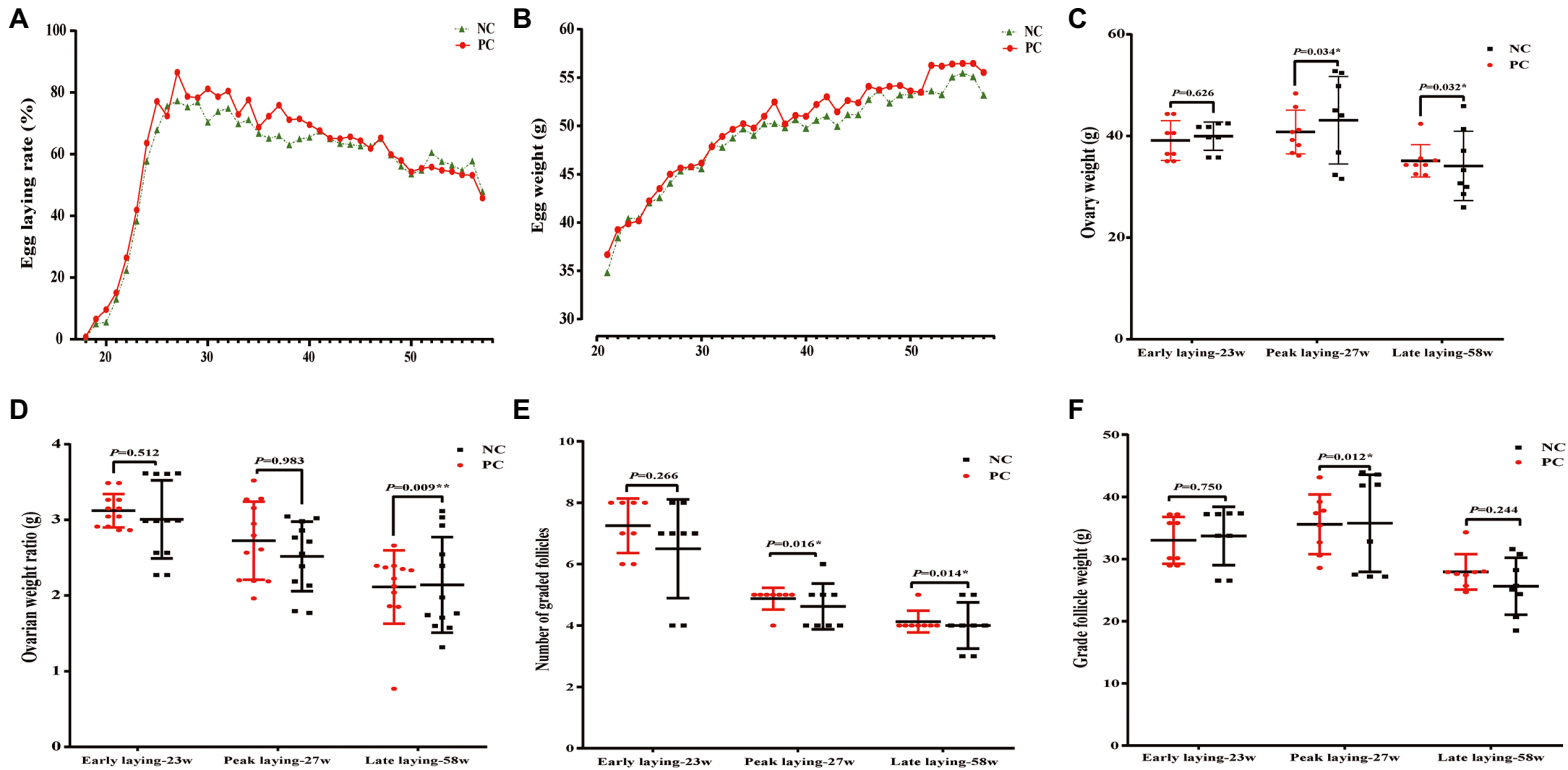
Effect of dietary supplementation of probiotics on performance, number and weight of follicles of laying hens at different laying period. **(A)** Lay rate; **(B)** Egg weight; **(C)** Ovary weight; **(D)** Ovarian weight ratio; **(E)** Number of grade follicles; **(F)** Grade follicles weight. Data present the mean ± SEM, *p < 0.05, **p < 0.01.

The laying rate of Tianfu green shell laying hens in each group were shown in Figure 1A. Compared with the NC group, dietary supplementation with probiotics significantly increased the laying rate of hens during the whole laying period (including early laying, peak laying and late laying) (*p* < 0.05). However, it is worth noting that after 52 weeks, the egg production rate of the PC group was slightly lower than that of the NC group, but the difference not significant (*p* > 0.05). Moreover, probiotic supplementation increased the average weekly egg weight, especially the average weekly egg weight at weeks 37, 41, 42, 48, 52 and 53 was significantly higher than that of the control group (*p* < 0.05) (Figure 1B), indicating that dietary probiotics can improve the egg weight of laying hens in the late laying period. The broken egg rate data are summarized in Table 2. Before 45 weeks of age, compared with the NC group, the egg breaking rate of PC group was significantly decreased, but after 46 weeks of age, the egg breaking rate was slightly increased.

**Table 2:** The effect of probiotics on broken egg rate.

Items	Treatments		SEM	*p* value
	PC	NC		
Broken egg rate, %				
Weeks 18–24	0.66^b^	1.02^a^	0.20	0.000
Weeks 25–31	0.84^b^	1.01^a^	0.11	0.000
Weeks 32–38	0.36^b^	0.83^a^	0.25	0.000
Weeks 39–45	0.64^b^	1.04^a^	0.22	0.025
Weeks 46–52	1.48	1.43	0.07	0.074
Weeks 53–58	2.45^a^	2.18^b^	0.16	0.023

PC, Dietary probiotics supplementary group; NC, Control group; SEM, stand error of the mean. ^ab^Values within a row with no common superscripts differ significantly (*p* < 0.05).

In addition, the ovarian weight and number of follicles of different sizes were determined. The results showed that probiotics had certain effects on the development of reproductive organs of laying hens. Compared with the NC group, the number of grade follicles in the PC group increased throughout the laying period, and the ovarian weight and graded follicles weight also increased in the late laying period (Figures 1C, E, F). However, the ovarian weight ratio decreased significantly in the late laying period (Figure 1D, *p* < 0.01). Meanwhile, as shown in Table 3, the weight and number of SYFs, SWFs and LWFs tended to increase with dietary probiotics supplementation during the whole laying cycle, but the difference was not significant (*p* > 0.05). However, the number of LYFs in the PC group were decreased at late laying period, but the difference was not significant (*p* > 0.05).

**Table 3:** The effect of probiotics on pregrade follicle weight and number in laying hens at different laying period.

Item	Time (week)	LYFs				SYFs				LWFs				SWFs			
		NC	PC	SEM	*P*	NC	PC	SEM	*P*	NC	PC	SEM	*P*	NC	PC	SEM	*P*
Number	23	0.50 ± 0.52	0.58 ± 0.67	0.588	0.737	6.25 ± 9.07	6.25 ± 4.75	7.085	1.000	2.75 ± 3.38	2.83 ± 3.01	3.134	0.950	9.25 ± 7.57	13.33 ± 9.32	8.564	0.252
	27	0.75 ± 0.62	1.42 ± 1.73	1.316	0.222	9.58 ± 4.56	10.67 ± 4.38	4.407	0.559	4.50 ± 3.75	6.33 ± 4.49	4.159	0.290	19.08 ± 10.62	22.75 ± 13.25	11.894	0.462
	58	0.92 ± 0.79	0.75 ± 0.62	0.702	0.572	7.67 ± 3.75	9.25 ± 4.57	4.170	0.364	5.83 ± 2.48	7.00 ± 2.82	2.669	0.294	15.08 ± 6.64	19.75 ± 11.98	9.771	0.251
Weight (g)	23	0.15 ± 0.16	0.20 ± 0.27	0.220	0.546	0.43 ± 0.75	0.44 ± 0.37	0.578	0.978	0.39 ± 0.44	0.41 ± 0.45	0.438	0.910	0.16 ± 0.17	0.24 ± 0.19	0.179	0.286
	27	0.45 ± 0.52	0.81 ± 0.73	0.648	0.177	0.87 ± 0.60	1.33 ± 1.01	0.845	0.188	1.04 ± 0.74	1.23 ± 1.10	0.921	0.635	0.92 ± 0.87	1.04 ± 0.84	0.839	0.729
	58	0.27 ± 0.22	0.28 ± 0.22	0.217	0.841	0.55 ± 0.32	0.69 ± 0.41	0.370	0.366	0.82 ± 0.43	1.02 ± 0.47	0.451	0.293	0.40 ± 0.23	0.43 ± 0.24	0.232	0.739

All data were expressed as mean ± standard deviation. PC, Dietary probiotics supplementary group; NC, Control group; SEM, stand error of the mean.

### Probiotics supplementary improved egg quality at different laying stages

Table 4 shows the effects of dietary probiotics supplementation on egg quality of laying hens at different laying stage. Compared with NC group, probiotics supplementation increased ESS, EW, AH, HU, yolk percentage and ESI during the whole laying period, especially in the late laying period (58w), the difference in ESS (*p* = 0.001) and ESI (*p* = 0.019) between the two groups was significant (*p* < 0.01, *p* < 0.05). The ET of the PC group was significantly higher than that of NC group at early laying (23w) and late laying (58w) (*p* < 0.01, *p* < 0.05), but lower than that of NC group at peak laying (27w), and the difference was not significant (*p* > 0.05). Moreover, compared with the NC group, YC in PC group was slightly increased and decreased at early laying and peak laying period, respectively, but not significant (*p* > 0.05).

**Table 4:** The effect of probiotics on the egg quality of laying hens at different laying period.

Item	Time (week)	PC	NC	*P* value
Egg shape index	21	1.27 ± 0.03	1.28 ± 0.03	0.692
	27	1.32 ± 0.03	1.31 ± 0.04	0.527
	52	1.36 ± 0.04^a^	1.32 ± 0.03^b^	0.019
Eggshell strength (Kg/cm^2^)	21	3.87 ± 0.36	3.59 ± 0.48	0.159
	27	3.84 ± 0.47	3.63 ± 0.93	0.531
	52	4.15 ± 0.32^a^	3.46 ± 0.47^b^	0.001
Eggshell thickness (um)	21	0.40 ± 0.02^a^	0.35 ± 0.01^b^	0.000
	27	0.33 ± 0.03	0.34 ± 0.02	0.482
	52	0.34 ± 0.03^a^	0.31 ± 0.02^b^	0.035
Egg weight (g)	21	39.11 ± 2.57	36.83 ± 2.44	0.057
	27	41.32 ± 2.03	40.25 ± 1.65	0.208
	52	56.36 ± 3.00	55.61 ± 2.17	0.531
Albumen height (mm)	21	6.34 ± 0.49^b^	6.83 ± 1.23^a^	0.015
	27	6.36 ± 0.45	6.16 ± 0.77	0.488
	52	7.68 ± 0.49	7.13 ± 0.71	0.058
Yolk color	21	13.00 ± 0.40	12.83 ± 0.33	0.312
	27	12.88 ± 0.49	13.04 ± 0.59	0.517
	52	12.00 ± 0.82^b^	13.40 ± 0.52^a^	0.000
Haugh unit	21	86.64 ± 3.24	88.85 ± 5.76	0.305
	27	86.34 ± 2.60	85.34 ± 6.49	0.657
	52	88.63 ± 2.54	85.49 ± 4.22	0.059
Yolk percentage (%)	21	26.57 ± 1.07	26.14 ± 1.88	0.535
	27	27.72 ± 0.63	27.19 ± 1.28	0.251
	52	33.56 ± 1.25	32.47 ± 1.48	0.092
Eggshell percentage (%)	21	14.00 ± 1.07	13.69 ± 1.00	0.509
	27	13.58 ± 0.91	12.97 ± 0.92	0.152
	52	12.07 ± 0.86	12.23 ± 0.58	0.617

All data were expressed as mean ± standard deviation. For the same line of data, different lowercase superscripts indicate significant differences (*p* < 0.05), no letters or the same letters indicate no significant differences (*p* > 0.05). PC, Dietary probiotics supplementary group; NC, Control group.

### Probiotics supplementary affected cholesterol and apolipoprotein levels in yolk

**Figure 2:**
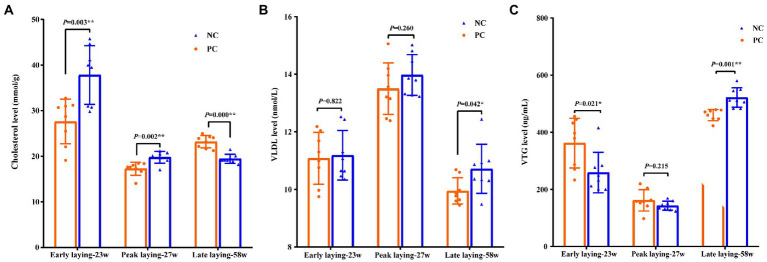
Effects of probiotic supplementation on lipid metabolism in serum and cholesterol content of laying hens in egg yolk at different laying period. **(A)** Cholesterol; **(B)** VLDL; **(C)** VTG. Data present the mean ± SEM, *p < 0.05, **p < 0.01.

To investigate the effects of probiotic supplementation on cholesterol and apolipoprotein, the contents of cholesterol, VLDL and VTG in yolk were measured. Compared with NC group, the level of cholesterol and VTG in yolk of PC group were significantly increased and decreased in early laying and peak laying period (*p* < 0.01), respectively, but were opposite in late laying period (Figures 2A, C). Moreover, the VLDL level of yolk in the PC group were decreased compared with the NC group during the whole laying period, and reached a significant level in the late laying period (*p* < 0.05, Figure 2B).

### Effects of probiotics on the serum hormone level

**Figure 3:**
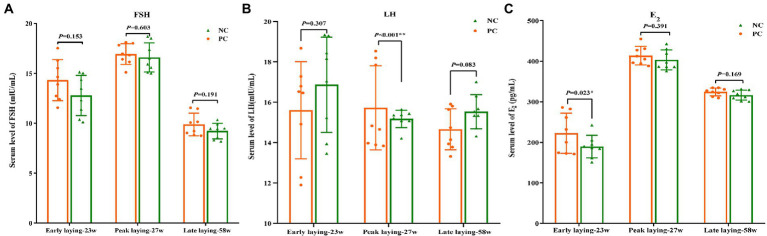
Effect of dietary probiotics on serum hormone levels of laying hens at different laying period. **(A)** Follicle-stimulating hormone (FSH); **(B)** Luteinizing hormone (LH); **(C)** Estradiol (E2). Data present the mean ± SEM, *p < 0.05, **p < 0.01.

The content of serum hormone can reflect the development and maturity of ovarian follicles of laying hens. As shown in Figures 3A, C, compared with the NC group, FSH and E_2_ levels in the PC group were increased during the whole laying period, and there was no significant difference between the two groups (*p* > 0.05) at other laying periods except that the E_2_ content in PC group was significantly higher than that in the NC group at early laying period (*p* < 0.05). Moreover, an increase in the serum concentration of LH was observed in the PC group compared to the NC one during the peak laying (*p* < 0.05), but decreased during the early laying and late laying period (Figure 3B).

### Probiotics supplementation reduced oxidative stress

**Figure 4:**
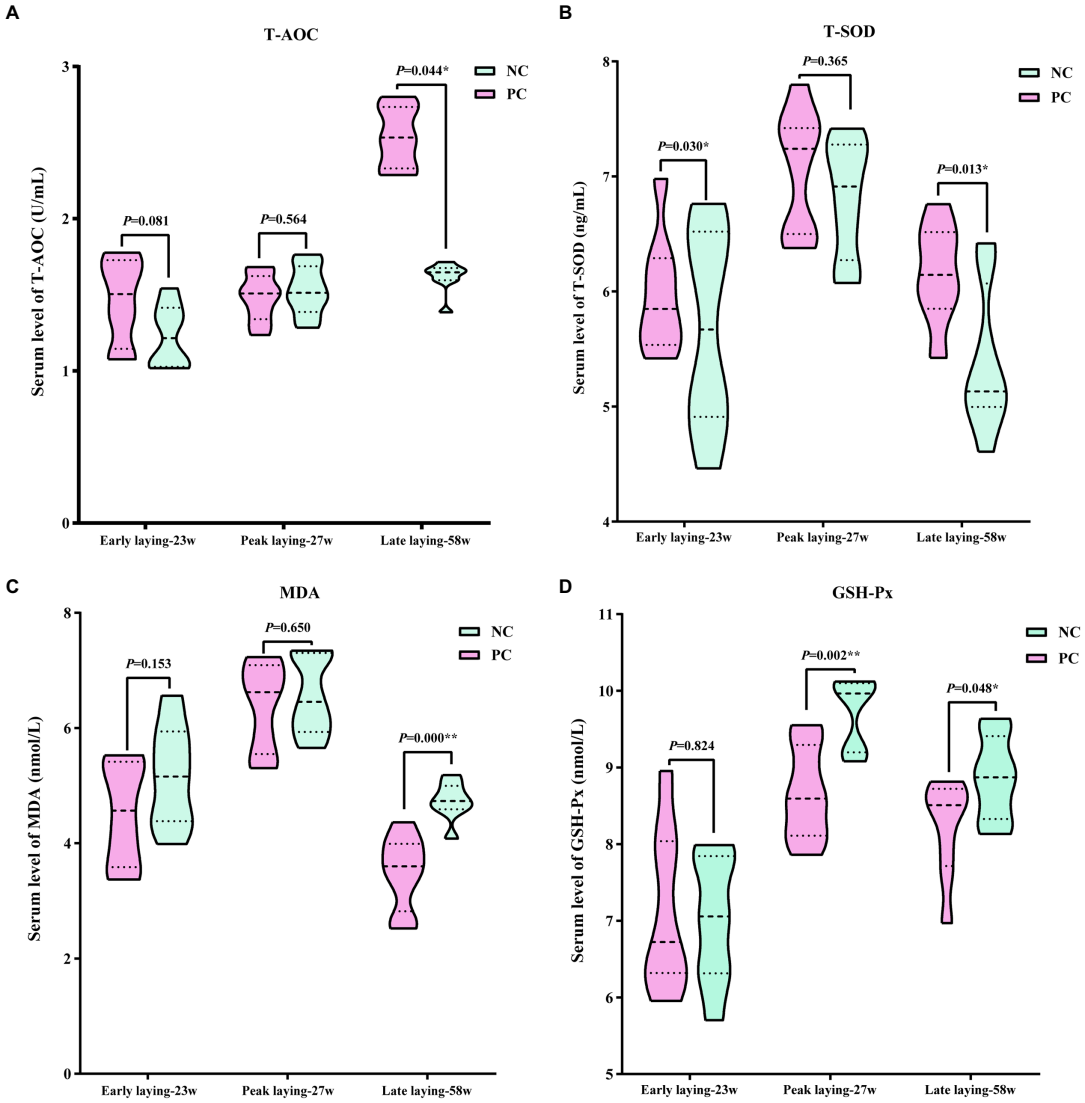
Effect of dietary probiotics on serum antioxidant index levels of laying hens at different laying period. **(A)** T-AOC; **(B)** T-SOD; **(C)** MDA; **(D)** GSH-Px. Data present the mean ± SEM, *p < 0.05, **p < 0.01.

Using serum data from laying hens at different laying period, Figure 4 presents the antioxidant stress status. The data indicated that compared with the NC group, probiotics supplementation increased T-AOC and T-SOD levels during the whole laying period, as well as reduced the level of MDA, especially at the late laying period, the difference between the two groups was significant (*p* < 0.05 or *p* < 0.01, Figures 4A–C). However, the serum GSH-Px level in PC group was slightly higher than that of NC group at the early stage of laying, but significantly lower than that of NC group at the peak and late stage of laying (*p* < 0.05, *p* < 0.01, Figure 4D).

### Probiotics supplement improved ovarian function by increasing the gene level associated with ovarian development

**Figure 5:**
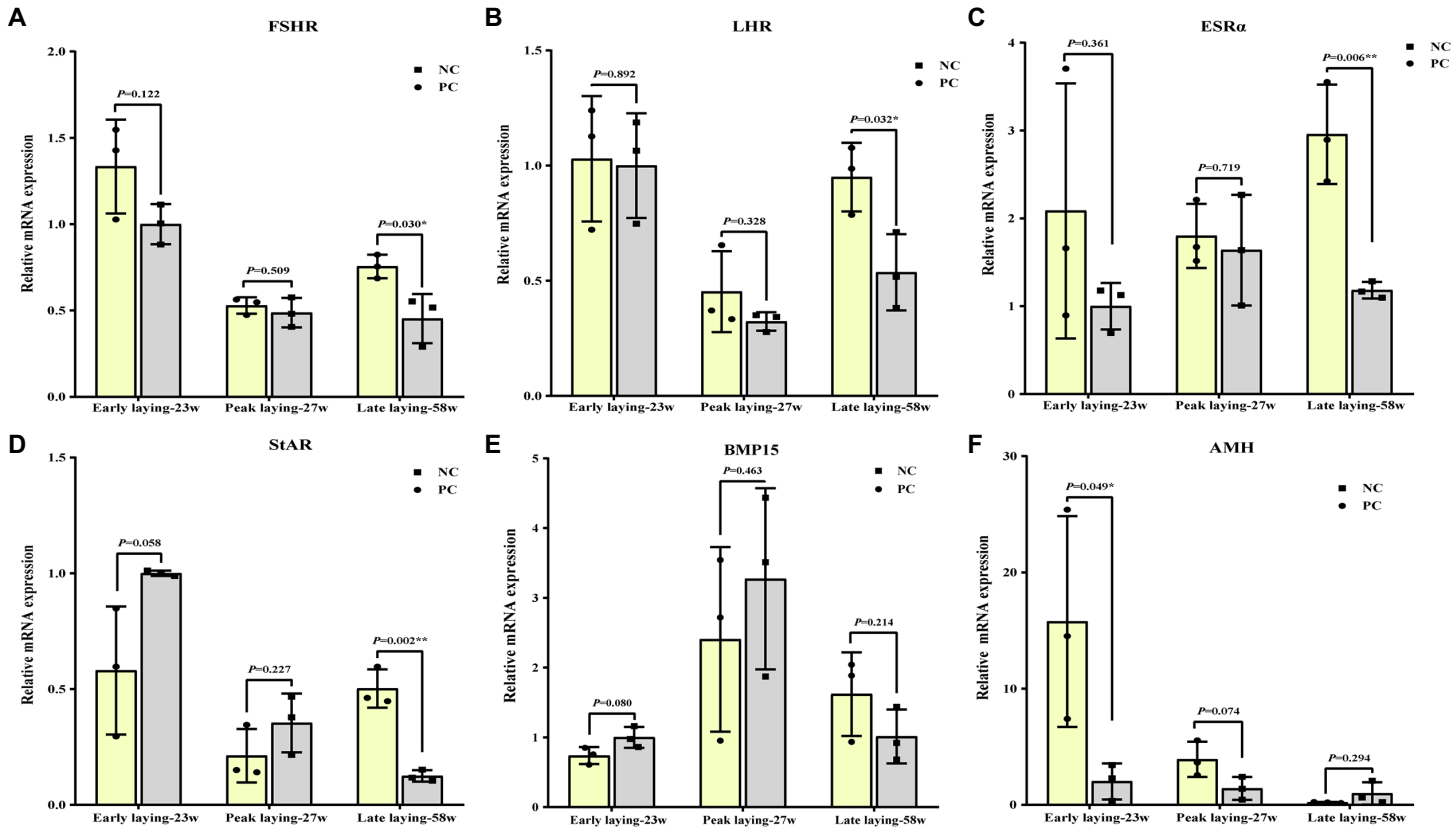
Effects of dietary probiotics on mRNA expression of genes related to ovarian development of laying hens at different laying period. **(A)** Follicle-stimulating hormone receptor (FSHR); **(B)** Luteinizing hormone receptor (LHR); **(C)** Estrogen receptors alpha (ERSα); **(D)** Steroidogenic acute regulatory protein (StAR); **(E)** Bone morphogenetic protein 15 (BMP15); **(F)** Anti-mullerian hormone (AMH). Data present the mean ± SEM, *p < 0.05, **p < 0.01.

To assess whether probiotics can improve ovarian development and function, we examined the expression of ovarian cytohormone receptor genes including *FSHR*, *LHR* and *ESRα*, ovarian *StAR*, *BMP15* and *AMH*. As shown in Figures 5A–C, the ovarian expression level of *FSHR*, *LHR* and *ESRα* in the PC group during the whole laying period were increased compared with the NC group, especially in the late laying period, reaching a significant or extremely significant level (*p* < 0.05, *p* < 0.01). After probiotics supplement, the expression levels of *StAR* and *BMP15* in ovary were significantly higher than those in the NC group at the late stage of laying (*p* < 0.05, Figures 5D, E), indicating an improvement in reproductive performance at the late stage. Interestingly, compared with the NC group, the expression level of *AMH* in the PC group increased significantly at the early laying and peak laying period (*p* < 0.05), but decreased at the late laying period (Figure 5F), as well as the *AMH* expression level in the ovarian tissues decreased with time in both the NC group and PC group.

### Probiotics supplement improved ovarian immune function

**Figure 6:**
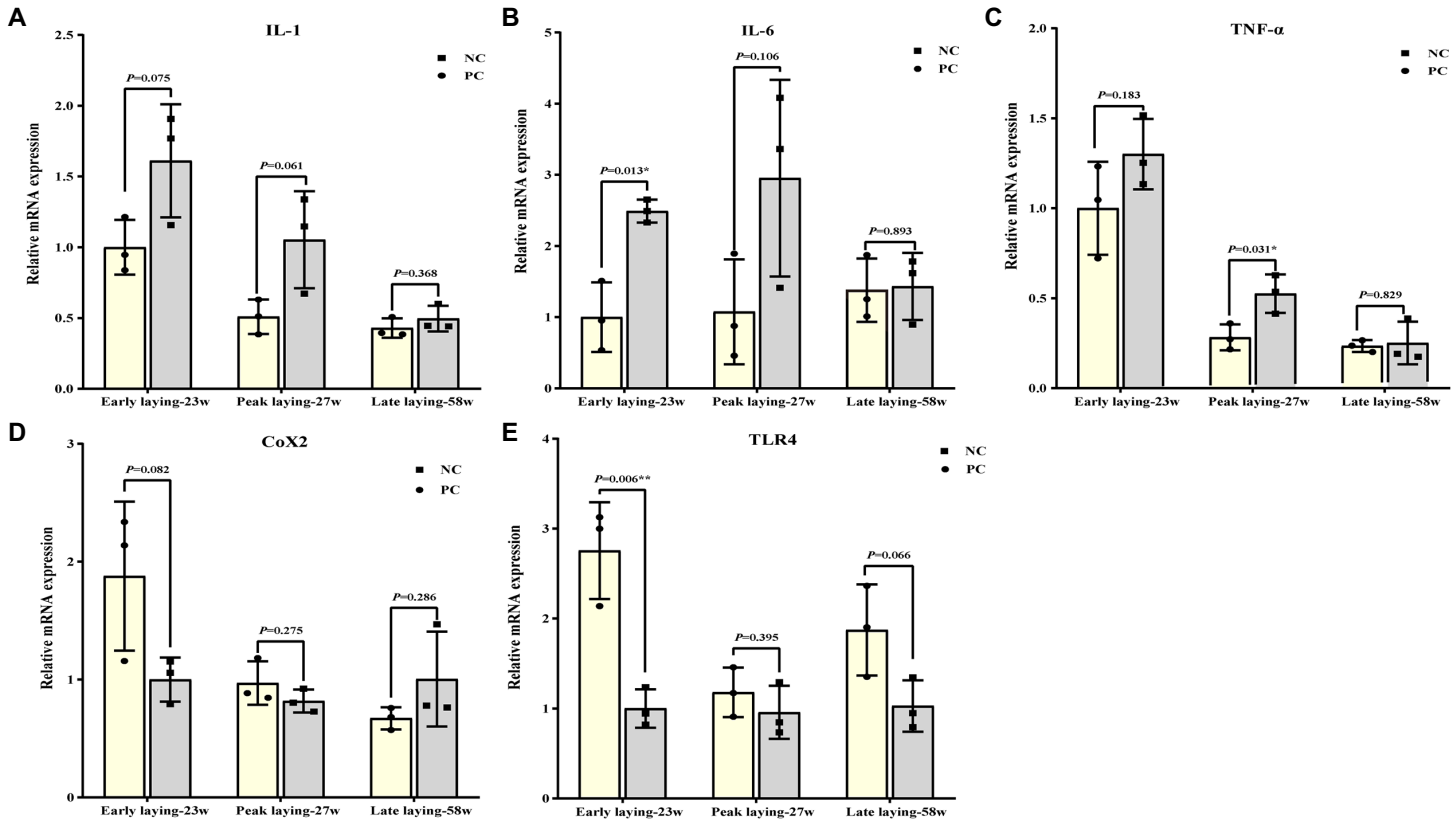
Effects of dietary probiotics supplementary on the mRNA expression of pro- and anti- inflammation related cytokines related genes in ovary at different laying period. **(A–D)** mRNA expression of proinflammatory cytokines including interleukin 1 (IL-1), interleukin 6 (IL-6), tumor necrosis factor-α (TNF-α), and cyclooxygenase-2 (COX2); **(E)** mRNA expression of anti-inflammatory cytokines related gene toll-like receptor 4 (TLR4). Data present the mean ± SEM, *p < 0.05, **p < 0.01.

Figure 6 summarized the relative mRNA expression of inflammation related cytokines in the ovarian. The expression level of proinflammatory cytokines such as *IL-1*, *IL-6* and *TNF-α* in ovary were decreased at three different laying stages due to the effect of probiotics as compared to NC group (Figures 6A–C), while the level of anti-inflammatory cytokines *TLR4* was increased (Figure 6E). There was no significant difference between the two groups at the late laying period, except that *TLR 4* and *IL-6* expression at the early laying period and *TNF-α* expression at the peak laying period were significantly different (Figures 6B, C, E, *p* < 0.05). Moreover, compared with the NC group, the expression level of *Cox2* in PC group was increased in the early laying and peak laying, as well as decreased in the late laying period, but the difference was not significant (Figures 6D, *p* > 0.05).

### Microbial composition in the cecal content

**Figure 7:**
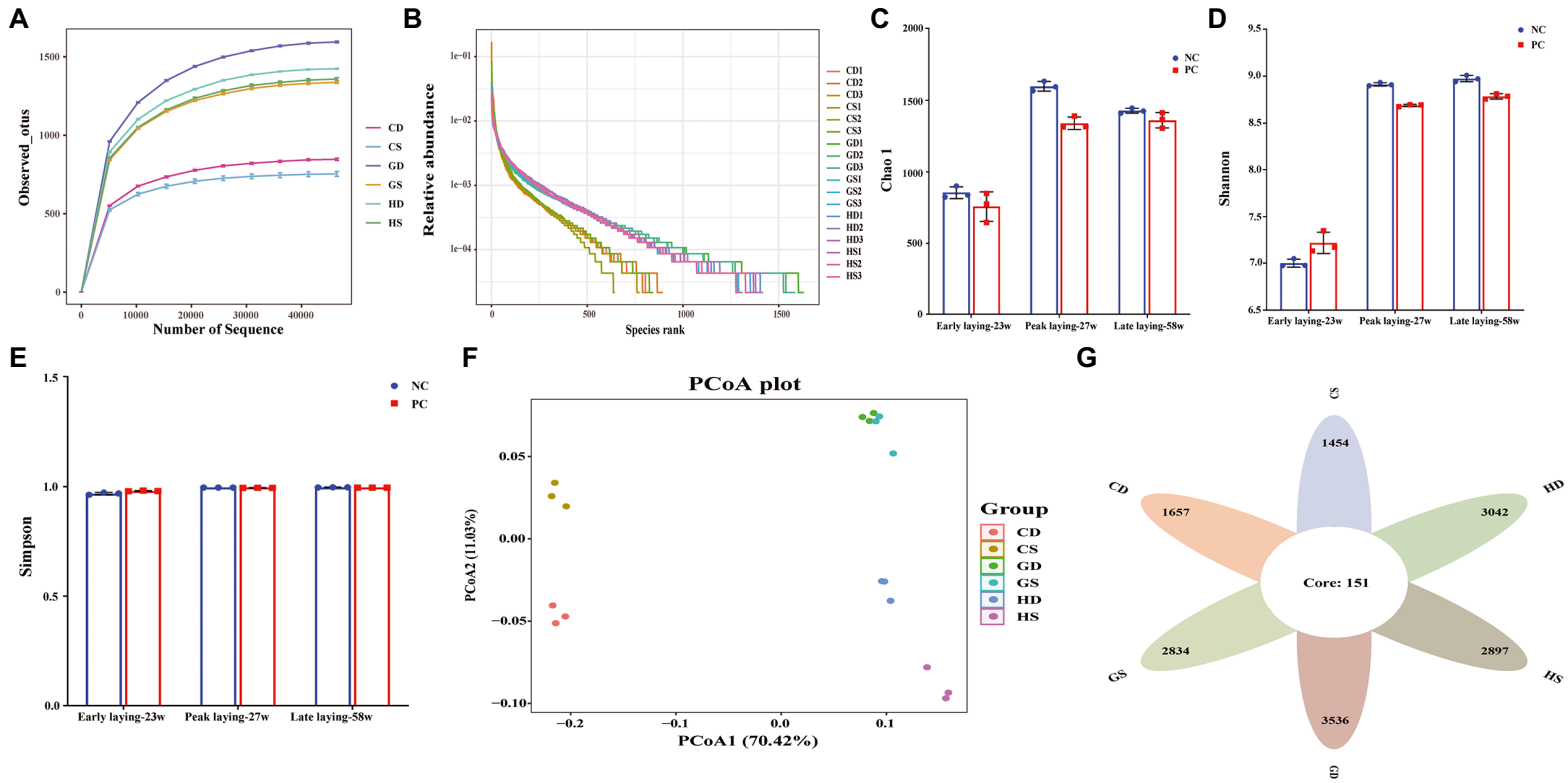
Effects of dietary probiotics supplementary on cecum microbiota diversity and composition in layers at different laying period. **(A)** Rarefaction curves. Sequences sampled represent sequencing reads that were grouped into operational taxonomic units (OTUs) based on their 97% similarity; **(B)** Abundance curve of species identified; **(C)** Chao 1 diversity index boxplot; **(D)** Shannon diversity index boxplot; **(E)** Simpson diversity index plot; **(F)** The principal coordinate analysis (PCoA) of the cecum microbiota based on unweighted UniFrac metric; **(G)** Venn diagram showing the number of control and probiotic-supplemented cecal microbial communities of layers at different laying stages sharing OTUs (97% similarity) and those that are unique.

To further investigate whether probiotics could affect intestinal microbiota at different laying stages, we analyzed the composition of cecal flora composition by 16S rDNA gene sequencing. A total of 1,170,991 V3-V4 16S rDNA effective sequences were obtained from the 18 samples, with an average of 65,055 sequences per sample. A total of 21,940 distinct operational taxonomic units (OTUs) were identified at the 97% identity level with high threshold identity and with an average of 1,219 OTUs for each group sample. The curves for the observed OUTs and species rank approached a plateau, suggested that the samples had sufficient sequence coverage to allow nearly all bacterial species in cecal samples to be identified (Figures 7A, B). Alpha diversity analysis revealed that Chao1 index were decreased in the PC group during the whole laying period (Figure 7C), and Shannon index in the PC group decreased in the peak and late laying period except that it was higher at the early laying period as compared with NC group; however, it had no difference between two groups (Figure 7D). Simpson indices showed that species evenness was the highest in the late laying period, reaching the maximum value of 1 (Figure 7E; Supplementary Table S1). Moreover, the principal coordinate analysis (PCoA) based on weighted unifrac distances further showed that there were differences in microbiota among all groups (Figure 7F). A Venn diagram showed that there were 151 OTUs common at different laying period in the caecum of laying hens. Additionally, 1,657 and 1,454 OUTs were uniquely present in the NC group and PC group at early laying period (23w), respectively; 3,042 and 2,897 OTUs were uniquely present in NC group and PC group at peak laying period (27w), respectively; 3,536 and 2,834 OTUs were uniquely present in the NC group and PC group at late laying period (58w), respectively (Figure 7G).

### Taxonomic composition of bacterial community

**Figure. 8:**
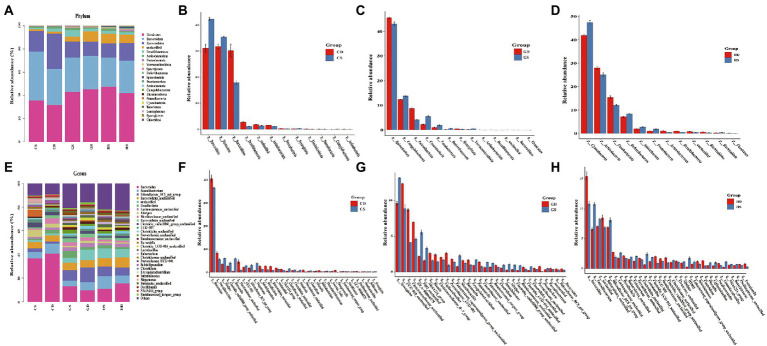
Relative abundance and significant difference of top 30 OTUs at the phylum and genus level in the cecal microbiota of laying hens at different laying stages. **(A)** The relative abundance of cecal microbiota at phylum level in laying hens; **(B)** Barplot difference analysis of cecal microbiota in phylum level of laying hens at early laying period; **(C)** Barplot difference analysis of cecal microbiota in phylum level of laying hens at peak laying period; **(D)** Barplot difference analysis of cecal microbiota in phylum level of laying hens at late laying period; **(E)** The relative abundance of cecal microbiota at genus level in laying hens; **(F)** Barplot difference analysis of cecal microbiota in genus level of laying hens at early laying period; **(G)** Barplot difference analysis of cecal microbiota in genus level of laying hens at peak laying period; **(H)** Barplot difference analysis of cecal microbiota in genus level of laying hens at late laying period.

Intestinal microbiota taxonomic analysis showed that the probiotic supplementation changed the composition of intestinal microbiota at different stages of the whole laying period. At the phylum level of the microbiota in the cecal, *Firmicutes Bacteroidota and Bacteroidetes* were the predominant bacteria from each group in this study, with relative abundance of 40.86, 30.77 and 16.93%, respectively. *Desulfobacterota* (2.73%) and *Actinobacteriota* (1.43%) were relatively lower in abundance, but they performed well across the whole experimental group. Other minor phyla such as *Proteobacteria* (0.43%), *Verrucomicrobiota* (0.33%), *Synergistota* (0.31%), *Deferribacterota* (0.29%), *Spirochaetota* (0.23%) and *Fusobacteriota* (0.14%) were also found (Figure 8A). We found a significant increase in relative abundance of *Firmicutes, Bacteroidota* and *Synergistota* in the PC group compared with the NC group at early laying (23w) (Figure 8B). Beyond that, compared with the NC group, *Bacteroidota*, *Desulfobacterota*, *Actinobacteriota, Verrucomicrobiota* and *Deferribacterota* in the PC group have increased significantly at peak laying (27w) (Figure 8C), and the relative abundance of *Firmicutes*, *Desulfobacterota* and *Actinobacteriota* also significantly increased at late laying (58w) (Figure 8D), but other bacterial phyla were decreased.

At the genus level, the dominant genera were *Bacteroidetes*, *Faecalibacterium*, *Rikenellaceae_RC9_gut_group*, and *Bacteroidales_unclassified* of all the groups (Figure 8E). Moreover, the relative abundance level of *Alistipes*, *Clostridia_vadinBB60_group_unclassified*, *Barnesiella, UCG-005, Rikenellaceae_RC9_gut_group* and *Megamonas* increased significantly after probiotics supplement at the early laying period (23w) (Figure 8F). Compared with the NC group, the relative abundance of *Bacteroides, Desulfovibrio* and *Prevotellaceae_unclassified* increased significantly, and the relative abundance of the *Rikenellaceae_RC9_gut_group*, *Faecalibacterium*, *Clostridiales_unclassified*, *Clostridia_vadinBB60_group_unclassified* and *Alistipes* decreased significantly at peak laying period (27w) (Figure 8G). It is noticeable that at late laying (58w), most cecal microorganisms in the PC group including *Faecalibacterium*, *Rikenellaceae_RC9_gut_group*, *Desulfovibrio, Eubacterium* and *Lactobacillus* increased dramatically (Figure 8H).

Changes in the cecum microbiome between NC group and probiotics supplement group (PC) at different laying period using linear discriminant analysis (LDA) effect size (LEfSe) was further compared based on the threshold of LDA score > 3. The results showed that there were 54, 80 and 75 dominant taxa of PC group in cecal content samples of Tianfu laying hens at the early, peak and late laying period, respectively, which could be used as potential biomarker bacteria (Supplementary Figure S1). It was observed that the PC group at the peak of laying period had more differential biomarkers, followed by the late laying period and the least at early laying period.

### Correlation analysis between the cecal bacteria

**Figure 9:**
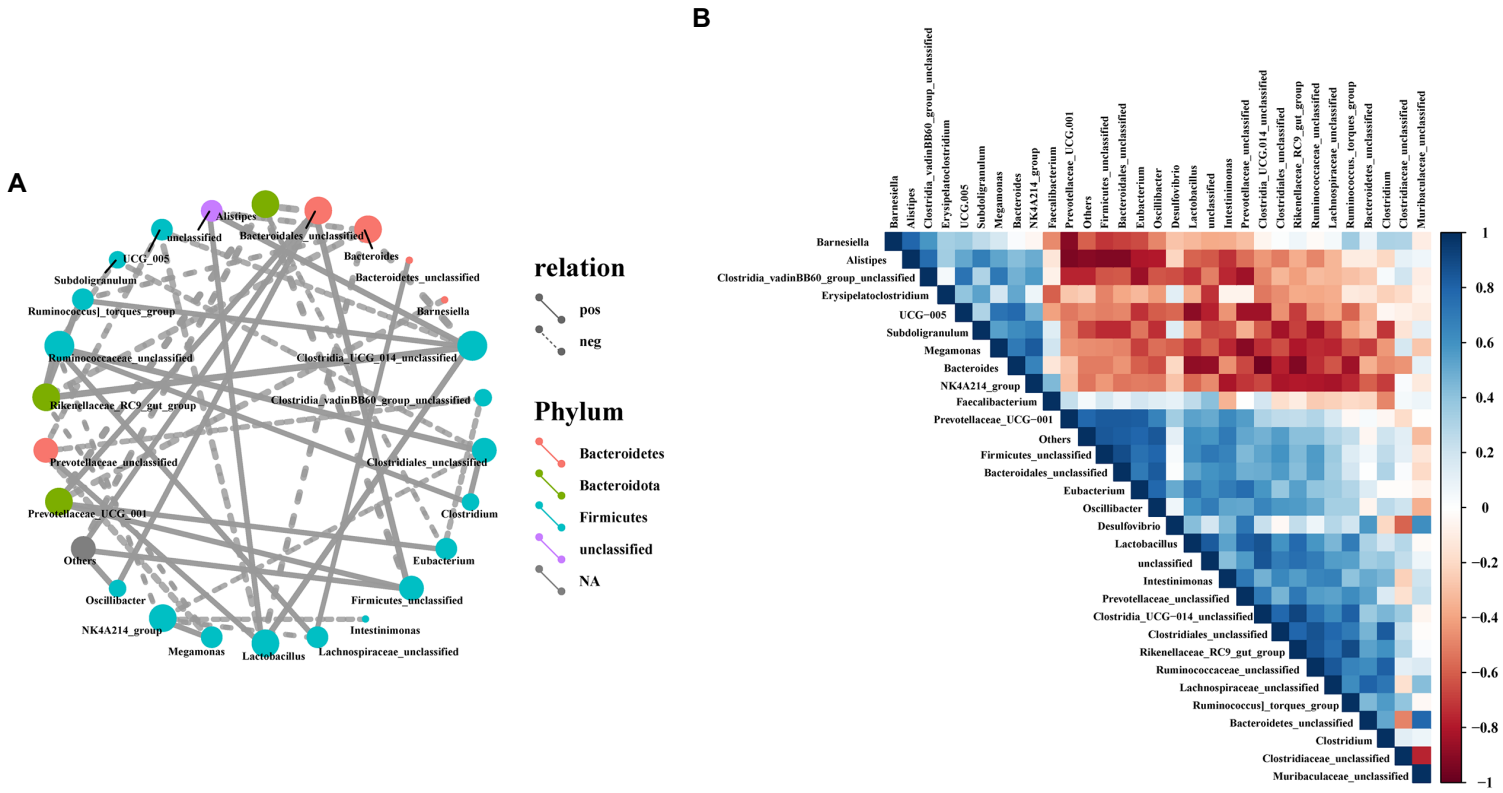
Correlation analysis of cecal microbiota in laying hens. **(A)** Network analysis at the genus level. Different nodes represent different dominant genera, the color of nodes indicates the phylum level species to which the species belongs, and the connection between nodes indicates the correlation between the two genera. A connecting line represents a significant correlation, whose width correlates with the strength of the correlation, while the line’s type indicated what type of interaction it is (solid line: positive correlation, dash line: negative correlation); **(B)** Heatmaps of Spearman correlation analyses among the abundance of top 30 gut microbiota in the cecum.

To investigate the possible interactions among members of the intestinal bacterial community in laying hens, we used Spearman correlation coefficient method to analyze the correlation between the top 30 microorganisms in genus level abundance. In this study, *Bacteroidetes_unclassified*, *Ruminococcaceae_unclassified*, *Clostridia_UCG-014_unclassified*, *Lactobacillus*, *Firmicutes_unclassified*, *Oscillibacter* and *Lachnospiraceae_unclassified* had a positive correlation with most bacteria (Figure 9A), but *Firmicutes_unclassified* and *Bacteroidetes_unclassified* had a significant negative correlation with *Muribaculaceae_unclassified* (Figure 9B). Furthermore, *Bacteroides*, *UCG-005*, *Alistipes*, *Subdoligranulum*, *NK4A214_group* and *Megamonas* had a significant negatively correlation with most bacteria, while *Megamonas* had a positively correlated with *NK4A214_group* (Figures 9A,B).

### Predictive functional profiling of microbial communities

**Figure 10:**
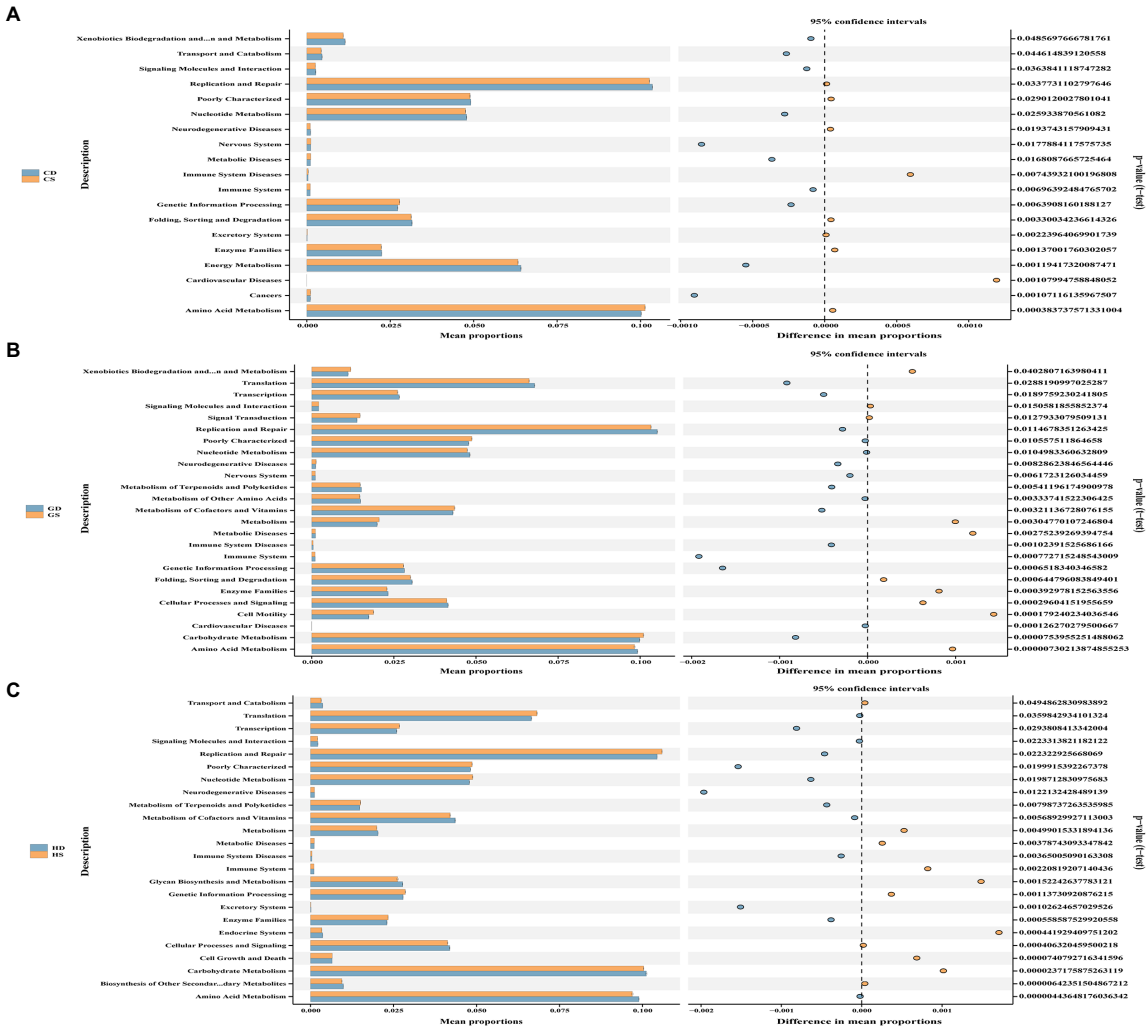
Functional analysis of microbial communities. PICRUSt2-predicted relative abundance of KEGG pathways (level 2) was compared between NC and PC groups of laying hens at different laying periods. **(A)** The early laying period (23w); **(B)** The peak laying period (27w); **(C)** The late laying period (58w). Welch’s correction was applied to the t test in order to analyze the differences between two groups at the same laying period. Statistical significance was determined by *p* < 0.01.

To further investigate the changes of cecal microbiota function in different laying stages after probiotics supplementation, we used PICRUSt2 technology to predict and analyze cecal microbiota function. Based on PICRUSt2 functional secondary classification results, the functional richness of cecal microbiota of hens at different laying stages was compared between the NC group and the PC group. The results shown that at the early laying period (23w), compared with NC group, PC group had extremely significantly increased gene clusters in amino acid metabolism, cardiovascular diseases, excretory system, immune system disease and metabolic diseases (Figure 10A; Supplementary Table S2; *p* < 0.01), and significantly increased gene clusters in cancer, genetic information processing, immune system and nervous system (Figure 10A; Supplementary Table S2; *p* < 0.05). Moreover, at the peak laying period (27w), 7 gene clusters of cell motility and carbohydrate metabolism in the PC group was extremely significantly higher than those in the NC group (Figure 10B; Supplementary Table S2; *p* < 0.01), while 3 gene clusters of signaling molecules and interaction, metabolism of cofactors and vitamins, and cardiovascular diseases in PC group were significantly higher than those in NC group (Figure 10B; Supplementary Table S2; *p* < 0.05). However, at the late laying period (58w), compared with the NC group, 9 gene clusters of the metabolism of terpenoids and polyketide, genetic information processing, enzyme families, immune system diseases, nucleotide metabolism, poorly characterized, replication and repair, transcription and translation were extremely significantly increased in PC group (Figure 10C; Supplementary Table S2; *p* < 0.01), as well as 1 gene clusters of cell growth and death enriched in the PC group (Supplementary Table S2; *p* < 0.05).

Meanwhile, we mapped 16S rRNA data to the KEGG pathway to predict the metabolic characteristics of gut microbiota at different laying period. At the early laying period (23w), compared with the NC group, probiotics supplementation significantly increased the terpenoid backbone biosynthesis, pyruvate metabolism, glycosyltransferases, glycine, serine and threonine metabolism, D-glutamine and D-glutamate metabolism, cell motility and secretion and benzoate degradation (Supplementary Figure S2A). Subsequently, the significant up of the tyrosine metabolism, two-component system, sulfur metabolism, pentose and glucuronate interconversions, inositol phosphate metabolism, naphthalene degradation, general function prediction only, fructose and mannose metabolism, bacterial motility proteins and bacterial chemotaxis in the PC group at the peak laying period (28w) (Supplementary Figure S2B). However, at the late laying period (58w), probiotics supplementation increased the pyrimidine metabolism, purine metabolism, peptidoglycan biosynthesis, peptidases, nucleotide excision repair, glycolysis/gluconeogenesis, glutathione metabolism, DNA repair and recombination proteins, bacterial secretion system and aminoacyl-tRNA biosynthesis compared to NC group (Supplementary Figure S2C).

### The influence of probiotics supplementation on microbiome characteristics at different laying period

BugBase was used to predict the bacterial composition of each group at different laying period. Nine potential phenotypes, including aerobic, anaerobic, contains mobile elements, facultatively anaerobic, forms biofilms, gram negative, gram positive, potentially pathogenic and stress tolerant were significantly predicted in the NC and PC group (*p* < 0.05), as shown in Supplementary Figure S3 and Supplementary Table S3. Among all the phenotypes, the PC group tended to have more anaerobic, gram-positive, stress tolerant, facultatively anerobic and containing mobile elements bacteria at early laying period (23w) and have more aerobic and gram-negative at peak laying period (27w). Additionally, the proportion of aerobic, facultatively anaerobic, forms biofilms, gram-positive and stress tolerant bacteria were observed to be significantly enriched in the PC group at the late laying period (58w). What is noteworthy is that the sum of potentially pathogenic bacteria was particularly lower in the PC group than in NC group at different laying time during the whole laying cycle (*p* < 0.01).

## Discussion

The reproduction performance of laying hens in the late laying period is directly related to the economic benefit of chicken farm. To our knowledge, this is the first study to add probiotics to the basic diet of Tianfu green shell laying hens in order to study its effects on egg quality, antioxidant capacity, immune function, and intestinal microflora at different laying stages. These results of this study can provide data reference and theoretical support for the application of probiotics in Tianfu green shell laying hens, and they have important implications for improving laying hen production performance at different laying stages, laying hen efficiency, and egg quality.

It is well known that maintaining egg shell quality and internal composition is a top priority for the laying hen’s industry. In the study, our findings showed that adding probiotics to the diet could increase the AH and HU at the peak and late laying period, and increased eggshell thickness and strength at late laying periods, which was similar to the results in laying hens in Mazanko et al. (2019). Also, Wang et al. (2021) reported that *Clostridium butyricum* can improve eggshell thickness and eggshell strength. Probiotics have been found to promote the growth of beneficial bacteria, and the proliferation of these microorganism ultimately leads to the accumulation of short-chain fatty acid (SCFAs) (Forte et al., 2016). Therefore, the improvement in eggshell quality may be related to the ability of probiotics to improve the serum calcium absorption and retention levels of laying hens, which promotes calcium deposition on shell glands (Attia et al., 2020). Conversely, some studies have found that probiotic supplementation has no effect on eggshell strength or thickness (Upadhaya et al., 2019; Wang et al., 2020). The reason may be related to the age and laying period of laying hens.

Ovary is the foundation of laying hens, and it is also one of the parts that commercial laying hen manufacturers pay special attention to, because changes in or destruction of their pathology affect the number and growth of follicles, which in turn affects egg production and quality, ultimately reducing economic benefits. In the present study, the number and weight of grade follicles, as well as the number of SYFs and LWFs in late laying period were improved by the addition of probiotics. However, little was known about the effects of probiotics on ovaria-related variables at different stages of laying hens for comparison with the current result. However, according to Lei et al. (2013), probiotics (*Bacillus licheniformis*) may increase serum FSH and E2 content in laying hens, which stimulated the growth and development of follicles. Meanwhile, Zhou et al. (2020) also found that the addition of probiotics (*Bacillus amyloliquefaciens*) would increase contents of FSH and E2, thus increasing the ovarian weight and promoting the growth and maturation of follicle. Thus, the addition of probiotics may regulate ovarian development by inducing FSH and E2 secretion, improving reproductive ability and follicles quality.

Oxidative stress occurs when there is a loss of balance between the antioxidant system and reactive oxygen species (ROS) as an output, which impairs reproductive performance and leads to tissue damage (Surai et al., 2019; Pisoschi et al., 2021). Therefore, antioxidant stress has become one of the important factors affecting the performance and egg quality of laying hens. There are pieces of evidence that probiotics could effectively mask the adverse effects of oxidative stress, and promote the activity of antioxidant enzymes (Deng et al., 2012). For instance, Zhan et al. (2019) investigated that effect of *Clostridium butyricum* on laying hens and found that the concentrations of GSH-Px, CAT and T-SOD were significantly increased in serum. Aluwong et al. (2013) showed that addition of yeast probiotics could significantly improve the GSH-Px activity of broilers. Also, Liao et al. (2015) revealed that dietary *Clostridium butyricum* supplementation could increase the activity of antioxidant enzymes and the concentration of major non-enzymatic antioxidant GSH in intestinal mucosa of broilers, while reducing the concentration of MDA. However, to our study, information is lacking on the effects of probiotics on Tianfu green shell laying hens’ antioxidation. Our present findings revealed that probiotics significantly increased serum T-AOC and SOD concentrations while decreasing the MDA content throughout the laying cycle, particularly in the late laying period. Overall, these findings suggest that complex probiotics could reduce oxidative stress in laying hens by stimulating enzyme components, thereby increasing laying rate and egg quality. It is suggested that these probiotics with antioxidant effect can be used as probiotic antioxidants in laying hen production, but the mechanism needs to be further investigated.

Furthermore, some studies have shown that probiotics or their cellular components can improve immune function in animals (Chen et al., 2022). For example, Zhao et al. (2022) revealed that dietary supplementation with the small peptides can reduce the proinflammatory factors including IL-6, IL-8 and IL-12 expression, thereby protecting the body from inflammatory damage. Also, Wang et al. (2021) showed that dietary addition of *Clostridium butyricum* and butyric acid glycerides decreased IL-6 content in jejunum of yellow-feathered breeder hens, while those of IL-4, IL-6, IL-1β and IgY were decreased by sodium butyrate. The present results showed that the dietary supplementation with probiotics upregulated the ovarian tissue of *TLR-4* expression while decreasing the mRNA level of proinflammatory cytokines (*IL-1*, *IL-6* and *TNF-α*) at three different time points during the laying period, implying that probiotics can regulate the ovarian immune function of laying hen. This is similar with the findings of Amevor et al. (2022) who reported that the dietary combination of quercetin and vitamin E decreased the mRNA levels of *1L-1β* and *IL-6* and increased the expression of anti-inflammatory cytokines (*IL-10*) in liver and testis, thus reduced the inflammatory response of the body improved the immune function. These results indicate that dietary probiotics can stimulate the local immune system in ovary of laying hens. The immunomodulatory effect of probiotics on laying hens was evidence in the reduced the expression level of proinflammatory cytokines. Therefore, we believe that the improvement of chicken immune status has a great promotion effect on chicken health, and is conducive to the improvement of egg production performance and egg shell and protein quality.

It is well known that the balance of gut microbiota is important for maintaining physiological and behavioral balance of chickens and is also essential for optimal growth, reproduction, health and welfare (Clavijo and Flórez, 2018). To better elucidate the effect of dietary probiotics on the intestinal microbiota of laying hens at different laying stages, 16S rRNA method is used to evaluate the effects. In this study, there was no significant difference in the diversity and richness of gut microbiota between the probiotic supplementation group and the control group at different laying stages. In line with previous studies, probiotics (*Bacillus licheniformis* DSM5749) had no significant effect on alpha diversity of cecal microorganisms of laying hens (Pan et al., 2022).

We also observed that at the phylum level, *Firmicutes*, *Bacteroidota* and *Bacteroidetes* accounted for the largest proportion of the total cecal microbial community of laying hens from each group, which was consistent with the results of previous studies (Zhang et al., 2021). During peak laying period, not only *Bacteroidota* increased, *Actinobacteriota*, *Verrucomicrobiota* and *Deferribacterota* also increased. By late laying period, the relative abundance of *Firmicutes*, *Desulfobacterota* and *Actinobacteriota* increased significantly, while other phyla have decreased. More detail indicated that at the genus level, the abundance of *Bacteroides* and *Prevotellaceae_unclassified* increased with the addition of probiotics at the peak laying period. Previous studies have shown that the *Bacteroidetes*, as a kind of probiotic bacteria, can improve the nutrient utilization and immunity of the host by increasing polysaccharide decomposition (Bai et al., 2013; Lee and Hase, 2014). Furthermore, *Prevotella* has an important role in the utilization of carbohydrates within the gut microbial ecosystem (Wang et al., 2017). This may explain why laying hens have greater feed intake and optimal performance at peaky laying period. Notably, the relative abundance of some beneficial bacteria such as *Faecalibacterium, Eubacterium* and *Lactobacillus* increased with the addition of probiotics at the late laying period. *Faecalibacterium* has important immune and metabolic functions, which was related to inflammation and possibly obesity (Balamurugan et al., 2010; Quevrain et al., 2016). It has been found that the abundance of *Faecalibacterium* in cecum and feces of chickens was associated with their health and growth (Singh et al., 2012; Wu et al., 2019). Meanwhile, Foditsch et al. (2015) also found that oral administration of *Faecalibacterium* to preweaning cows did improve weight gain and reduce diarrhea. Additionally, *Eubacterium* is also known to plays a critical role in regulating inflammation, immune responses, and maintaining the integrity of the intestinal barrier (Mukherjee et al., 2020). Therefore, we hypothesized that the improvement of ovarian immune function of laying hens by probiotics in the late laying period might be related to the changes of these microbiota levels. The exact mechanism needs to be further determined.

Notably, the bacterial composition predicted by BugBase demonstrated that probiotics supplementation significantly increased the proportion of aerobic, facultatively anaerobic, forms biofilms, gram-positive and stress tolerant bacteria, while decreased the proportion of potentially pathogenic bacteria at the late laying period. Previous studies have shown that biofilm formation is a protective growth pattern that against the threat of antibiotics and harsh environments (Davies, 2003; Hall and Mah, 2017), thus explaining why the probiotic supplementation can enhance the immune function of hens at late laying period. However, these results are based on BugBase predictions and need to be verified experimentally.

## Conclusion

In conclusion, dietary supplementation with probiotics was effective in improving egg quality, promoting antioxidant capacity and immune function, and benefiting follicle development and cecal microflora of laying hens at different laying stages, especially at the late laying period (Supplementary Figure S4).

## Data availability statement

The original contributions presented in the study are publicly available. This data can be found at: NCBI, PRJNA874899.

## Ethics statement

The animal study was reviewed and approved by Institution Animal Care and Use Committee of Sichuan Agricultural University, Sichuan, China. Written informed consent was obtained from the owners for the participation of their animals in this study.

## Author contributions

HX and YW conceived and designed this study. YXL, DL, CY, YJ, ZH, ZZ, and RD performed the experiments. HX and YW analyzed the data and wrote the manuscript. YXL, DL, and CY assisted in the 16S rRNA gene sequencing data analysis and the phenotypic data analysis. XZ, YZ, YT, QZ, and YPL collected all samples of this study. XZ, YPL, and YW edited the paper. YW obtained the funding. All authors reviewed and approved the final manuscript.

## Funding

This study was supported by the 14th Five-year Plan for Breeding Program in Sichuan (2021YFYZ0007).

## Conflict of interest

The authors declare that the research was conducted in the absence of any commercial or financial relationships that could be construed as a potential conflict of interest.

## Publisher’s note

All claims expressed in this article are solely those of the authors and do not necessarily represent those of their affiliated organizations, or those of the publisher, the editors and the reviewers. Any product that may be evaluated in this article, or claim that may be made by its manufacturer, is not guaranteed or endorsed by the publisher.

## Footnotes

^1^ http://www.arb-silva.de/
