# Supplementary material for: Probiotic mediated intestinal microbiota and improved performance, egg quality and ovarian immune function of laying hens at different laying stage
Source: Front Microbiol. 2023 Jan 24;14:1041072. doi: 10.3389/fmicb.2023.1041072 (PMC9902371; doi:10.3389/fmicb.2023.1041072)
Supplement: Supplementary file 1 [file Data_Sheet_1.docx]

**SUPPLEMENTARY INFORMATION**

**Figure S1** Key phylotypes of cecum microbiota of laying hens at different laying stages. (A) Cladogram generated from LEfSe analysis. The radiations of different circle layers from inside to outside represent seven taxonomic levels of phylum family species respectively, and the node size corresponds to the average relative abundance of species. Yellow nodes indicate species that are not significantly different between the two groups, and red, blue and green areas indicate the abundance of different species in each group. (B) Linear discriminant analysis (LDA) scores derived from LEfSe analysis. The x-axis represents the LDA score (Log 10 score) and the Y-axis represents the species with significant differences (LDA SCORE>3).

**Figure S2** Predicted metabolic profile of the cecum microbiome at different laying period. (A) The early laying period (23w); (B) The peak laying period (27w); (C) The late laying period (58w). By using Welch’s t-test (*p*<0.05), significance was determined between the NC and PC group. The colored circles represent 95% confidence intervals calculated using Welch’s inverted method.

**Figure S3** Compared the bacterial composition predicted by BugBase between the control group and the probiotic supplementation group at different laying stages. (A) Aerobic; (B) Anaerobic; (C) Contains mobile elements; (D) Facultatively anaerobic; (E) Forms biofilms; (F) Gram-negative; (G) Gram-positive; (H) Potentially pathogenic; (I) Stress tolerant.

**FIGURE S4** Mechanism diagram of effect of probiotics on Tianfu Green shell laying hens.
