# Supplementary figures and images for: Probiotic mediated intestinal microbiota and improved performance, egg quality and ovarian immune function of laying hens at different laying stage
Source: Front Microbiol. 2023 Jan 24;14:1041072. doi: 10.3389/fmicb.2023.1041072 (PMC9902371; doi:10.3389/fmicb.2023.1041072)

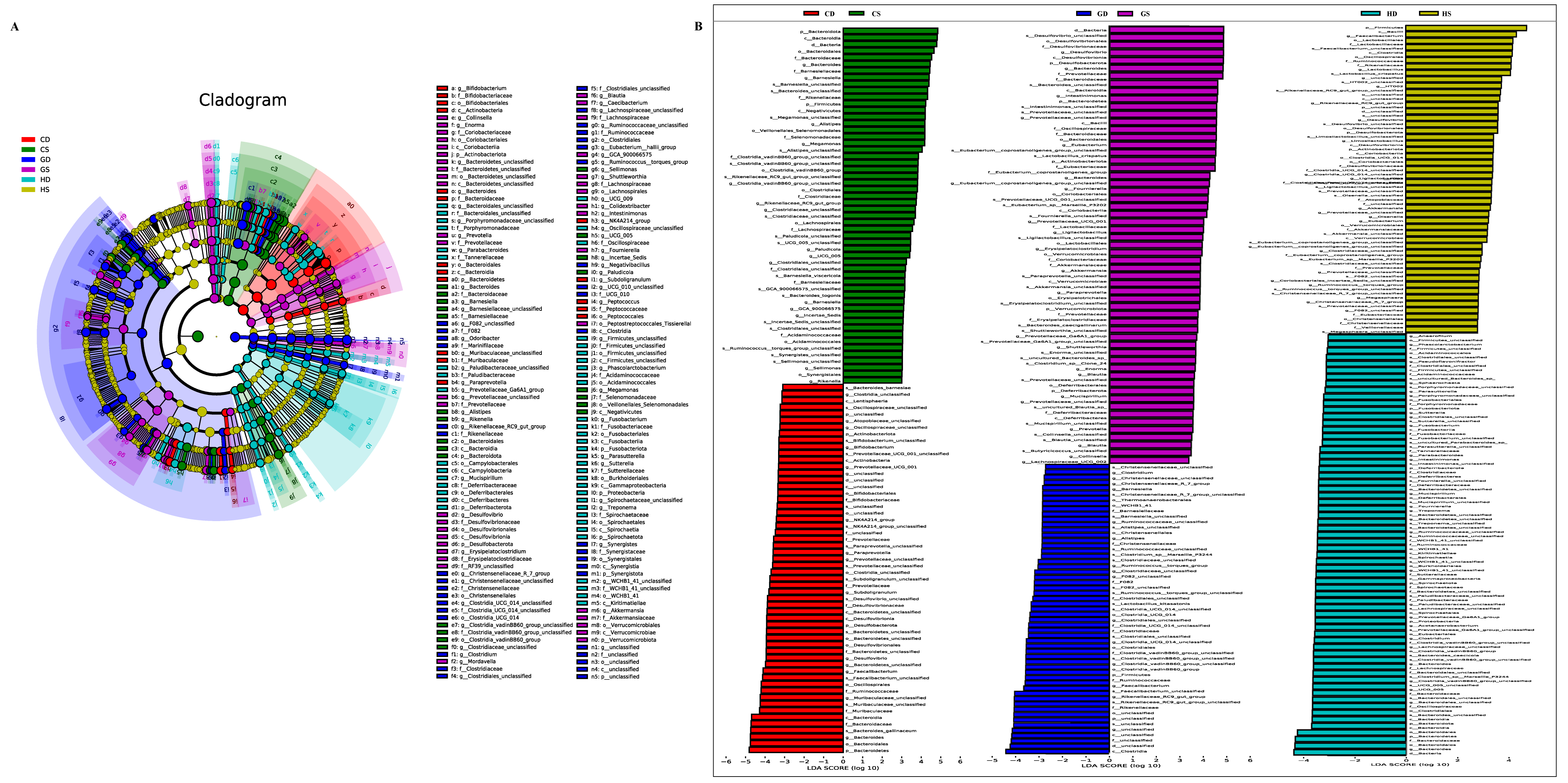

Supplement: Supplementary file 2 [file Image_1.TIF]

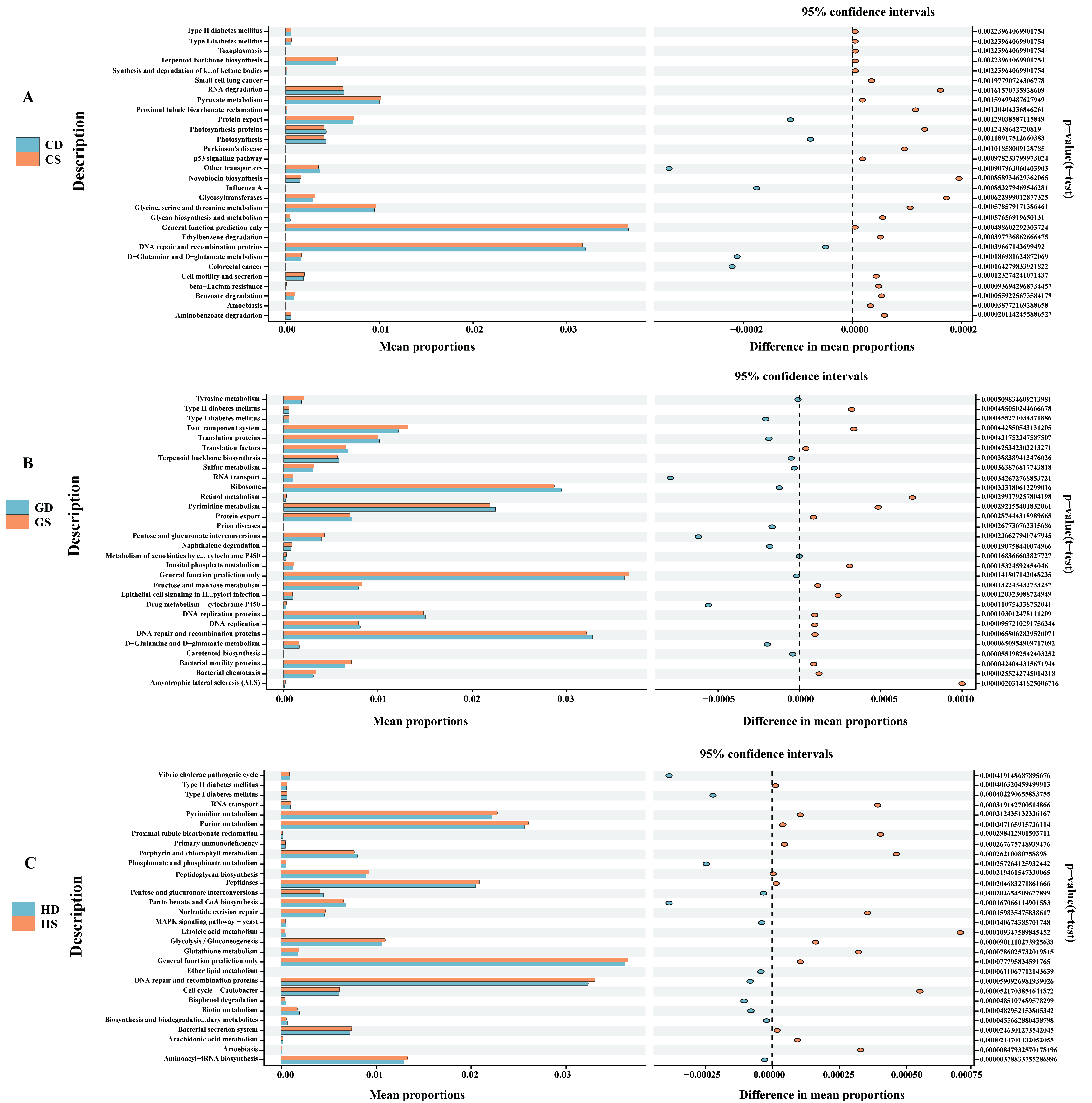

Supplement: Supplementary file 3 [file Image_2.TIF]

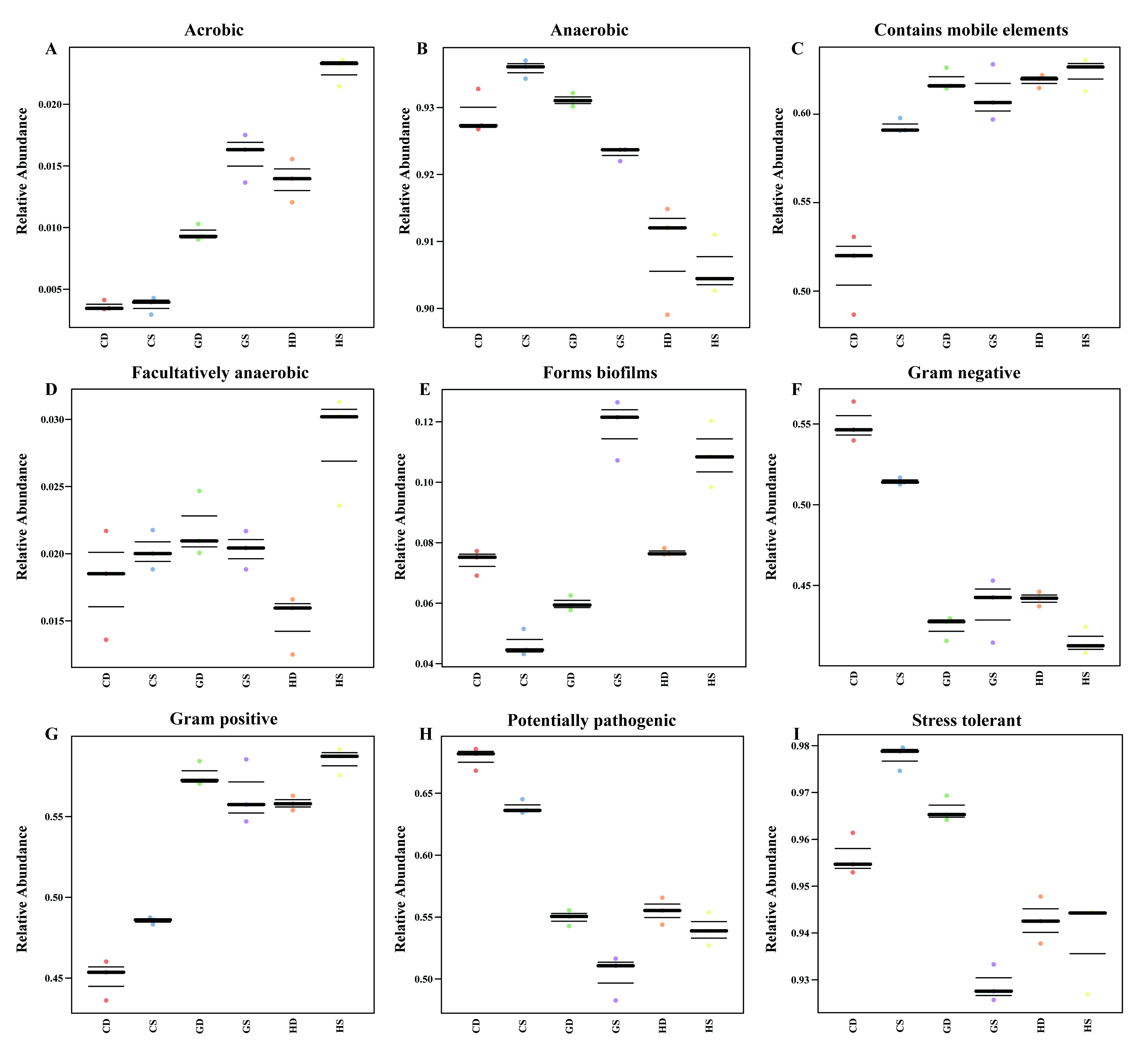

Supplement: Supplementary file 4 [file Image_3.TIF]

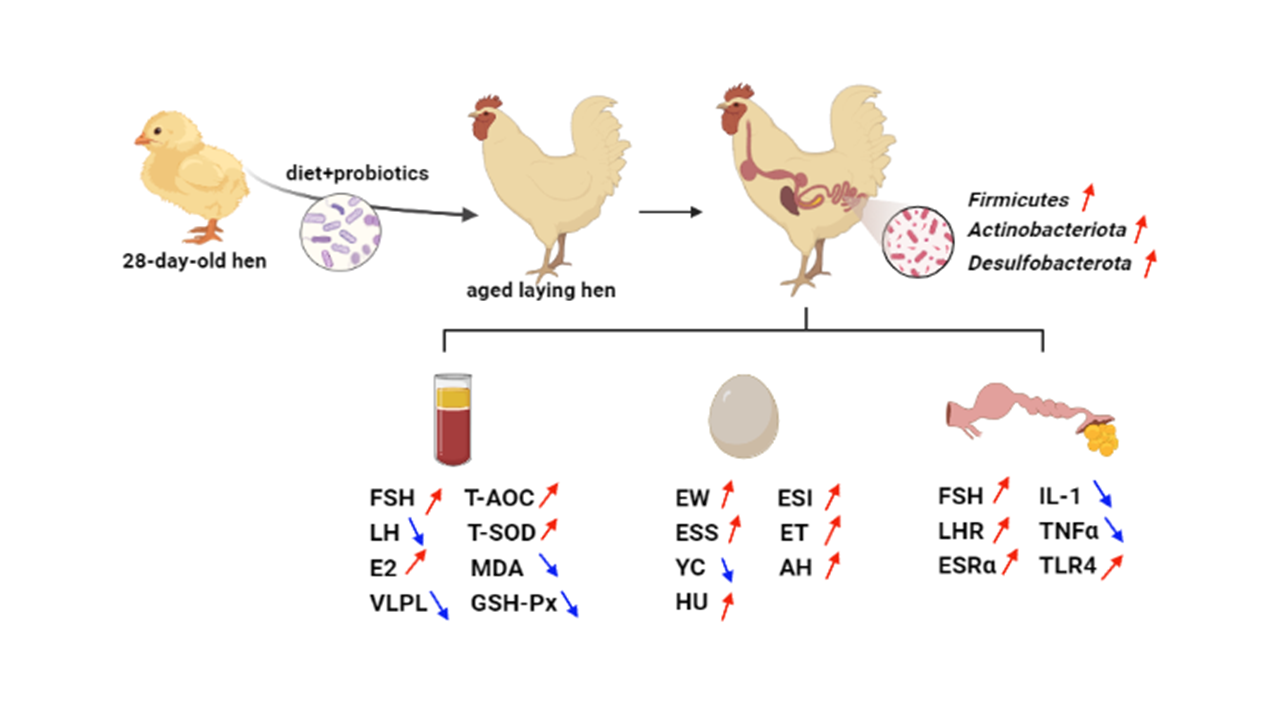

Supplement: Supplementary file 5 [file Image_4.TIF]
